# Supplementary material for: Persistence of Tembusu Virus in Culex tritaeniorhynchus in Yunnan Province, China
Source: Pathogens. 2023 Mar 21;12(3):490. doi: 10.3390/pathogens12030490 (PMC10058924; doi:10.3390/pathogens12030490)
Supplement: Supplementary file 1 [file pathogens-12-00490-s001.zip › pathogens-2250815-supplementary.pdf]

Table S1. Primers used for amplification of the ORF of YN2020-20 strain.

Primer[a]	Position[b]	Sequence (5′ -3′)
T1-f	37-57	TTGGAGTAGTGCGTGTGAACG
T1-r	3033-3056	AGCTCAGGTCACTGTGCACAGCTC
T2-f	2906-2931	TGGAATAGTATGAAGATTGAAGACTT
T2-r	5792-5814	GTGGTTATCACAAAATCCCATTC
T3-f	5668-5690	CCTGATAGAGCTTGGAATTCTGG
T3-r	8563-8586	CAAATTCTTCACCCTATCAGCGAC
T4-f	8223-8248	ATGGCCGAGGAATGGCTAGCTAGGG
T4-r	10938-10967	AGCCACACTTTCGGCGATCTGTGCCAAGTG

[a] f, forward primer; r, reverse primer.

[b] Position refer to the genome of BYD-1 (GenBank accession number JQ920420).

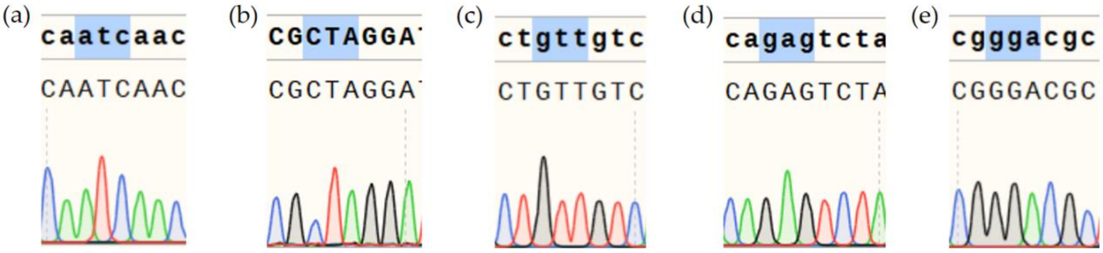

Figure S1. (a), (b), (c), (d), and (e) are the alignment of amino acid loci E-358, NS1-113, NS4A-89, NS4B-D/E/N/C22S, and NS5-638 by sanger sequencing, respectively. The first row is the result of high-throughput sequencing, the second row is the result of singer sequencing. The blue area indicates an amino acid site.

Table S2. Sequence information used for analysis in this study.

Reference TMUV'S	Year of collection	Location	Host	Accession No.
MM 1775	1955	Malaysia	Mosquito	JX477685
Sitiawan	2000	Malaysia	avian	JX477686
DK/TH/CU-DTMUV2007	2007	Thailand	avian	MF621927
flavivirus	2010	China	avian	NC015843
HB2010	2010	China	avian	MN649262
SD2010	2010	China	avian	MN649260
FX2010	2010	China	avian	MH414568
SH201001	2010	China	avian	KY623443
SH201002	2010	China	avian	KY623442
FX2010	2010	China	avian	KY623434
ZJ2010B2	2010	China	avian	KY623422

HB201002	2010	China	avian	KY623418
HB201001	2010	China	avian	KY623417
FQ-C1	2010	China	avian	KX977555
ZJQT17	2010	China	avian	KX977552
AH-F10	2010	China	avian	KM102539
TMUV	2010	China	avian	KF192951
LD 2010	2010	China	avian	KC990544
LC 2010	2010	China	avian	KC990543
BZ 2010	2010	China	avian	KC990540
Shandong1	2010	China	avian	JX965381
JS/2010	2010	China	avian	JX273153
duck/WR/China/2010	2010	China	avian	JX196334
FJMH220	2010	China	avian	JQ928189
GS-PT-7	2010	China	avian	JQ627864
CK-SD-11	2010	China	avian	JQ627862
XHZD/2010	2010	China	avian	JQ595407
ZJ GH-2	2010	China	avian	JQ314465
ZJ 407	2010	China	avian	JQ314464
CJD05	2010	China	avian	JF926699
JS804	2010	China	avian	JF895923
ZJ-6	2010	China	avian	JF459991
BYD-1	2010	China	avian	JF312912
YY5	2010	China	avian	JF270480
TMUV-YY1Du	2010	China	avian	AB917088
TC2B	2011	China	avian	MH764605
HB201102	2011	China	avian	KY623427
JS201101	2011	China	avian	KY623426
JS201102	2011	China	avian	KY623425
SD201147	2011	China	avian	KY623424
SD201120	2011	China	avian	KY623423
SD201151	2011	China	avian	KY623421
HB201101	2011	China	avian	KY623416
NCLT230	2011	China	avian	KX977554
ZJLJS71	2011	China	avian	KX977553
FJFQ300	2011	China	avian	KX977551
GD06	2011	China	avian	KX977550
FS-2011	2011	China	avian	KX686578
SDSG	2011	China	avian	KJ740746
GX 2011	2011	China	avian	KC990542
DU/CH/LSD/110128 F10	2011	China	avian	KC800808
Du/CH/LSD/110128	2011	China	avian	KC136210
JM	2011	China	avian	JN811559
FS	2011	China	avian	JN811558
GX2012	2012	China	avian	MN649264

HB201214	2012	China	avian	KY623420
HB201217	2012	China	avian	KY623419
D1921/1/3/MY	2012	Malaysia	avian	KX097990
D1977/1/MY	2012	Malaysia	avian	KX097989
YN12193	2012	China	Mosquito	KT607936
YN12115	2012	China	Mosquito	KT607935
xz 2012	2012	China	avian	KM188953
DTMUV-AH2011	2012	China	avian	KJ958533
Du/CH/LSD/110128 P90	2012	China	avian	KJ782380
Du/CH/LSD/110128 P70	2012	China	avian	KJ782379
Du/CH/LSD/110128 P50	2012	China	avian	KJ782378
Du/CH/LSD/110128 P30	2012	China	avian	KJ782377
df-2	2012	China	avian	KJ489355
TMUV-SDHS	2012	China	avian	KF826767
zc-1	2012	China	avian	KF557894
lq-1	2012	China	avian	KF557893
WFZ 2012	2012	China	avian	KC990545
FX 2012	2012	China	avian	KC990541
SDMS	2012	China	Mosquito	KC333867
WJ-1	2012	China	avian	JX549382
TMUV-JSGo	2012	China	avian	AB917090
TMUV-WZDu	2012	China	avian	AB917089
BJ2013	2013	China	avian	MN649265
HD1-2013	2013	China	avian	KX686577
PY-2013	2013	China	avian	KX686576
HD2-2013	2013	China	avian	KX686575
DK/TH/CU-1	2013	Thailand	avian	KR061333
GX2013C	2013	China	avian	KP861859
TMUV-SH001	2013	China	avian	KP742476
GX2013G	2013	China	avian	KM275941
GX2013E	2013	China	avian	KM275940
CQW1	2013	China	avian	KM233707
SX1	2013	China	avian	KM066945
AHQY	2013	China	avian	KJ740748
SDLC	2013	China	avian	KJ740747
SDXT	2013	China	avian	KJ740745
GX2013H	2013	China	avian	KJ700462
AH2014	2014	China	avian	MN649267
SD14	2014	China	avian	MH748542
HZ-2014	2014	China	avian	KX686580
PY-2014	2014	China	avian	KX686573
MC	2014	China	avian	KX452096
GD2014	2014	China	avian	KU323595
G23	2014	China	avian	KT239021

GDHD2014-3	2014	China	avian	KT159713
TMUV-JS06	2014	China	avian	KR869106
DTMUV/CH/2014	2014	China	avian	KP096415
GX2015	2015	China	avian	MN649263
HN2015	2015	China	avian	MN649261
DK/TH/CU-1	2015	Thailand	Mosquito	MH460536
zjYY150903	2015	China	avian	MF522176
zjYY150901	2015	China	avian	MF522174
zjYY150902	2015	China	avian	MF522175
DTMUV JS-S2/2015	2015	China	avian	KY810819
DTMUV JS-S1/2015	2015	China	avian	KY810818
JS-L1/2015	2015	China	avian	KY626659
JS201502	2015	China	avian	KY623441
ZJ201508	2015	China	avian	KY623440
ZJ201506	2015	China	avian	KY623439
ZJ201507	2015	China	avian	KY623438
ZJ201505	2015	China	avian	KY623437
ZJ201503	2015	China	avian	KY623436
ZJ201501	2015	China	avian	KY623435
ZJ201502	2015	China	avian	KY623433
ZJ201504	2015	China	avian	KY623432
AH201501	2015	China	avian	KY623431
AH201502	2015	China	avian	KY623430
AH201504	2015	China	avian	KY623429
JS201501	2015	China	avian	KY623428
HZ3-2015	2015	China	avian	KX686579
HZ2-2015	2015	China	avian	KX686574
HD-2015	2015	China	avian	KX686572
HZ4-2015	2015	China	avian	KX686571
HZ1-2015	2015	China	avian	KX686570
GDLH01	2015	China	avian	KT824876
HB2016	2016	China	avian	MN649266
SDDZ	2016	China	avian	MH764606
NMCF	2017	China	avian	MH764607
DTMUV/QY/17	2018	China	avian	MT447092
GA	2018	China	avian	MK907880
P4	2019	China	avian	MZ574097
SCS01	2019	China	avian	MW143073
WS12101	2019	China	avian	MT951413
DTMUV/Goose/CHN/2019/AQ-19	2019	China	avian	MT708901
H	2019	China	avian	MT108702
CHN-YC	2019	China	avian	MN966680
CHN-JL	2019	China	avian	MN966679

TP1906	2019	China	Mosquito	MN747003
GL	2019	China	avian	MK889501
Y	2019	China	avian	MK542820
NTUC225/20	2020	China	avian	MW821486
BZ0162	2020	China	avian	MT951412
**YN2020**	**2020**	**China**	**Mosquito**	**OQ238827**
